# Establishment of a Japanese version of the Sick, Control, One Stone, Fat, and Food (SCOFF) questionnaire for screening eating disorders in university students

**DOI:** 10.1186/s13104-021-05549-0

**Published:** 2021-04-16

**Authors:** Yutaka Hosoda, Toshiyuki Ohtani, Hisashi Hanazawa, Mami Tanaka, Hiroshi Kimura, Noriaki Ohsako, Tasuku Hashimoto, Osamu Kobori, Masaomi Iyo, Michiko Nakazato

**Affiliations:** 1 Department of Psychiatry, School of Medicine, International University of Health and Welfare, 4-3, Kozunomori, Chiba, 286-8686 Japan; 2 Safety and Health Organization, Chiba University, Chiba, Japan; 3 Training Division for School Health Nursing Techers, Faculty of Education, Chiba University, Chiba, Japan; 4 Center for Forensic Mental Health, Chiba University, Chiba, Japan; 5 Department of Psychiatry, Graduate School of Medicine, Chiba University, Chiba, Japan; 6 Department of Psychology, International University of Health and Welfare, Narita Hospital, Narita, Japan

**Keywords:** Eating disorders, Screening questionnaire, Early detection, Bodyweight loss

## Abstract

**Objective:**

This study aimed to validate the Sick, Control, One stone, Fat, and Food (SCOFF) questionnaire in relation to the Eating Disorders Examination Questionnaire (EDE-Q) and to examine the appropriateness of a question concerning weight loss among Japanese university students. The psychometric properties of the two Japanese versions were determined among 649 Japanese college students. The original version (SCOFF-O) employed the original item 3, whereas the revised version (SCOFF-2.5) modified the item to “Have you recently lost more than 2.5 kg within three months?” Validity was tested relative to EDE-Q.

**Results:**

The test–retest reliabilities of SCOFF-O and SCOFF-2.5 were 0.52 and 0.57, while the correlations of SCOFF-O and SCOFF-2.5 with EDE-Q were *r* = 0.53 and *r* = 0.56. The sensitivity and specificity of SCOFF-O were 65.2 and 89.7, and those of SCOFF-2.5 were 69.5 and 86.5, respectively. There were significant correlations between the question concerning losing 2.5 kg and the EDE-Q subscales. The Japanese version of SCOFF-2.5 is an appropriate tool for the early screening of eating disorders among Japanese university students.

## Introduction

The emergence of eating disorders (EDs) in Asia over the past three decades is multifactorial and includes causes related to pathological and cultural transformation [1]. A study by Hudson, Hiripi, Pope, and Kessler [2] found that the onset of EDs coincides with the age of college entry, while Volpe et al. [3] found that most of those with anorexia nervosa (AN) tended to be early adolescents. About 80% of college students with significant clinical symptoms of EDs do not receive appropriate care [4].

Therefore, the early detection of EDs in adolescents and undergraduate college students is of concern. A feasible assessment tool is needed to screen large populations for EDs or those at risk of developing EDs. Given the time constraints facing school health care, the screening tool needs to be brief, validated, and easy to remember.

The Eating Attitude Test-26 (EAT-26) [5] is most frequently used as a screening tool in Japan. However, the sensitivity and specificity of the EAT-26 make it a poor screening tool for eating disorder pathology in Japan [6]. The EDE-Q was developed to screen and evaluate EDs severity [12] and was used as the reference standard for evaluation of SCOFF quesitonaire [8].

The Sick, Control, One stone, Fat, and Food (SCOFF) questionnaire, consisting of five questions, was developed as a simple screening instrument for EDs [7]. Leung et al. [8] confirmed the sensitivity of SCOFF in 100% of women aged 18–40 years who were already diagnosed with AN or bulimia nervosa. The validity of the translated versions of the SCOFF questionnaire has been established in several countries, including some countries in Asia [8]. In Japan, the SCOFF and EAT-26 questionnaires were validated via clinical samples [9]. They suggested that the question concerning bodyweight loss (Question 3) should be modified because the question only detect the recent extreme body weight loss but not detect the low body weight. Some questions regarding the adaptation of the original version of SCOFF for the Japanese population are based on differences in the average body mass index (BMI: weight [kg]/height [m]^2^) of young Japanese people. The percentage of women and men aged 20–29 years who were (at the time of data collection) underweight (BMI < 18.5 kg/m^2^) was 21.7% and 9.0%, respectively [10, 11].

In the original SCOFF questionnaire, Question 3 refers to a weight loss of one stone over three months. In the current study, the Japanese version of the SCOFF questionnaire uses “6.35 kg” instead of “one stone.” The average BMI of 20-year-old Japanese women is 20.8 kg/m^2^ [10] and the loss of one stone would reduce their BMI to 18.2 kg/m^2^. In the present study, item 3 was modified to, “Have you recently lost more than 2.5 kg within three months?” This item 3b was subsequently included in the Japanese version of the SCOFF questionnaire in addition to the original question (item 3a). For early detection among young people, losing 2.5 kg would be a useful measure among the average 20-year-old woman, where it would result in an average BMI score of 19.7 kg/m^2^ (from 20.8 kg/m^2^), whereas for men of the same age, losing 2.5 kg would result in a decrease in average BMI from 21.8 kg/m^2^ to 20.9 kg/m^2^. We hypothesize that the questionnaire asking about losing 2.5 kg (SCOFF-2.5) would be more sensitive than the SCOFF-O (original version).

The first aim of this study was to verify the reliability and validity of both the Japanese SCOFF-O and the SCOFF-2.5 questionnaires relative to the Japanese version of EDE-Q among university students. The second aim of this study was to examine the appropriateness of the question concerning body weight loss in the SCOFF questionnaire.

## Main text

### Methods

#### Overview

An online survey of Chiba University students was administered between 2016 and 2017 as part of an online mental health checkup. Participants were asked to complete the Japanese version of the SCOFF and EDE-Q questionnaires and to provide demographic variables, including body height and weight.

#### Participants

A total of 649 college students (272 women, 377 men) with a mean age of 22.37 years (*SD* = 3.28) participated in this study. An additional 82 students (43 women, 38 men, and 1 unknown) with a mean age of 22.22 years (*SD* = 2.67) took part as a measure of the test–retest reliability of SCOFF-O and SCOFF-2.5. All participants could speak and understand Japanese fluently.

#### Japanese versions of the SCOFF Questionnaire

The original SCOFF questionnaire was translated into Japanese by four experts in EDs, back-translated into English, and subsequently approved by the original author. [7] The items of the Japanese version of the SCOFF questionnaire include:

1. Do you make yourself sick (vomit) because you feel uncomfortably full?

2. Do you worry that you have lost control over how much you eat?

3a. Have you recently lost more than 6.35 kg within three months?

3b. Have you recently lost more than 2.5 kg within three months?

4. Do you believe yourself to be fat when others say you are too thin?

5. Would you say that food dominates your life?

The total score of the SCOFF questionnaire (with items 1, 2, 3a, 4, and 5) is referred to as the original questionnaire (SCOFF-O). The total score of items 1, 2, 3b, 4, and 5 are referred to as SCOFF-2.5.

#### The Japanese version of the eating disorders examination questionnaire

The EDE-Q was developed to screen and evaluate EDs severity [12]. The Japanese version of the EDE-Q consists of 36 questions on the presence of EDs symptoms during the preceding 28 days based on four subscales: Dietary Restraint, Eating, Weight, and Shape Concern. Each item was rated on a seven-point Likert scale, with higher scores indicating more severe psychopathologies of EDs [13]. According to a study of university students in the USA, the cutoff point for global EDE-Q scores was ≥ 3 [14].

#### Procedure

The study was approved by the Ethics Committee of Chiba University. After reading about the purpose of the study, participants gave their informed consent. It took approximately 15 min to complete the questionnaire package. To examine test–retest reliability, SCOFF and EDE-Q were re-administered at two- to four-week intervals.

### Statistical analysis

Data were analyzed using SPSS statistics version 24.0 software (IBM Corporation, Armonk, NY, USA). The reliability of the SCOFF questionnaire was tested with interclass correlation coefficients and internal consistency measures using Cronbach’s alpha. Concurrent validity was analyzed by its correlations with the EDE-Q. [14].

### Results

#### Reliability testing of SCOFF-2.5 and SCOFF-O

To examine test–retest reliability, the Japanese SCOFF questionnaire and EDE-Q were tested at two- to four-week intervals. Fifty-eight out of 82 students (36 women, 22 men) with a mean age of 22.03 years (*SD* = 2.28) completed the questionnaires twice. The average weight, height, and BMI scores of the women were 50.96 kg (*SD* = 5.94), 158.86 cm (*SD* = 5.40), and 20.18 kg/m^2^ (*SD* = 2.07), and those for men were 65.23 kg (*SD* = 8.89), 171.94 cm (*SD* = 5.36), and 22.00 kg/m^2^ (*SD* = 2.15). The test–retest reliability of the SCOFF-O and SCOFF-2.5 questionnaires and the EDE-Q were 0.52 (95% CI: 0.31, 0.69) and 0.56 (95% CI: 0.36, 0.69). The internal consistencies of the SCOFF-O, SCOFF-2.5, and EDE-Q subscales ranged from 0.41. to 0.42, 0.40 to 0.46, and 0.58 to 0.93, respectively.

#### Internal consistency and validity of the japanese SCOFF questionnaire

Complete data regarding the SCOFF-O and SCOFF-2.5 questionnaires were obtained from 649 students, of which 58.0% were men (*n* = 377), and 41.9% were women (*n* = 272). The mean age of the students was 22.37 (*SD* = 3.28). The average weight, height, and BMI of the women were 51.54 kg (*SD* = 6.34), 158.02 cm (*SD* = 5.30), and 20.63 kg/m^2^ (*SD* = 2.24), and that for the men was 63.55 kg (*SD* = 12.13), 171.56 cm (*SD* = 6.13), and 21.55 kg/m^2^ (*SD* = 3.72), respectively; 12.3% of participants were underweight (*n* = 80; men = 40; women = 40), while 9.5% (*n* = 62; men = 40; women = 12) were overweight (> 25 kg/m^2^). The average weight and height at each age in this study were close to those reported in Statistics of Japan (e-State, Statistics of Japan, n.d.). Of the participants, 12.4% (37 men, 44 women) were SCOFF-O-positive (scoring two or more), while 15.4% (52 men, 51 women) were SCOFF-2.5-positive. Table 1 shows the breakdown of the answers.

**Table 1:** Number of yes/no responses for each item of the SCOFF questionnaire

Item no	Questions	Yes (n)	No (n)	Yes (%)
1	Do you make yourself sick because you feel uncomfortably full?	43	606	6.63
2	Do you worry you have lost control over how much you eat?	149	500	22.96
3a	Have you recently lost more than 6.35 kg within three months?	5	644	0.77
3b	Have you recently lost more than 2.5 kg within three months?	69	580	10.63
4	Do you believe yourself to be fat when others say you are too thin?	33	616	5.08
5	Would you say that food dominates your life?	80	569	12.33

As for content validity, both SCOFF-O and SCOFF-2.5 were moderately correlated with EDE-Q and its subscales (Table 2). The sensitivity and specificity of the SCOFF-O were 65.2% and 89.7%, when the EDE-Q global score (cutoff ≥ 3) was used as a reference for standard EDE-Q scores among undergraduate students. [14] The positive and negative predictive values were 21.7% and 98.3%, respectively. The sensitivity and specificity of SCOFF-2.5 with the EDE-Q global score (used as the reference standard) were 69.5% and 86.5%. The positive and negative predictive values were 18.3% and 98.4%, respectively.

**Table 2:** Pearson’s product moment correlation coefficient between the SCOFF, item 3a or 3b and the EDE-Q

	SCOFF-O	SCOFF-2.5	Item 3a	Item 3b
	*r*	*r*	*r*	*r*
Dietary restraint	0.39**	0.44**	0.07 *n.s*	0.27**
Shape concern	0.45**	0.47**	0.06 *n.s*	0.22**
Eating concern	0.55**	0.55**	0.00 *n.s*	0.20**
Weight concern	0.42**	0.45**	0.06 *n.s*	0.24**
Global score	0.52**	0.55**	0.06 *n.s*	0.28**

The original version of the SCOFF (SCOFF-O) included item 3a: “Have you recently lost more than 6.35 kg within three months?” The modified version of SCOFF (SCOFF-2.5) included item 3b: “Have you recently lost more than 2.5 kg within three months?”

*EDE-Q* Eating Disorders Examination Questionnaires; **p < 0.01

#### The relevance of the weight loss question to Japanese students

The answer “yes” for item 3b (losing 2.5 kg) was observed among 10.6% of respondents, which is more compatible than the 0.77% “yes” response rate for item 3a (losing 6.35 kg) (Table 1). There were significant correlations between the question concerning “losing 2.5 kg” and the subscale and global scores of EDE-Q (*r* = 0.20, 0.28; p < 0.01). Conversely, there were no significant correlations between the question concerning “losing 6.35 kg” and either the subscale or global scores of the EDE-Q (Table 2).

### Discussion

In Hong Kong (Leung et al., 2009) and Sweden (Hansson, Daukantaité, & Johnsson, 2015), 26.9% and 11.1% of secondary school students were SCOFF-positive [15]. The results of the present study (12.4% for SCOFF-O and 15.4% for SCOFF-2.5.) are similar to the approximate values from these reports. The internal consistencies for SCOFF-O and SCOFF-2.5 were inadequate (0.41 to 0.42 and 0.40. to 0.46, respectively), similar to reports from Hong Kong [8] because of the small number (five) of questions and the binary yes/no answers of the SCOFF questionnaire [16].

To detect Japanese adolescents at risk for EDs, a new question (item 3b, losing 2.5 kg) was included. The answer “yes” for item 3b (losing 2.5 kg) was registered among 10.6% of participants, which is more compatible than the 0.77% observed response rate for item 3a (losing 6.35 kg), with the rate (6.1%) of the Swedish version of SCOFF.^15^ The significant correlation between item 3b and EDE-Q subscales and global scores suggests that, as a screening tool for EDs, the inclusion of the question concerning 2.5 kg weight loss in the SCOFF may be more appropriate for early detection among Japanese university students.

## Conclusion

Both the original SCOFF and the revised SCOFF 2.5 questionnaires indicate a significant correlation with their global EDE-Q scores. Specifically, the question concerning losing 2.5 kg showed a significant correlation with their global scores of the EDE-Q.

SCOFF-O and SCOFF-2.5 showed good levels of specificity, while SCOFF 2.5 showed better levels of sensitivity than SCOFF-O. In particular, the version with the question concerning the 2.5 kg bodyweight loss is an appropriate tool for early screening of EDs among Japanese university students.

## Limitations

The participants were not diagnosed using structured interviews. The present study modified item 3 based on the average weight and height of adolescents in Japan. Future studies should test the SCOFF questionnaire among other generations with younger participants.

## Data Availability

The datasets generated and/or analysed during the current study are available in the Toshiyuki Ohtani repository (otani@chiba-u.jp).

## Abbreviations

AN: Anorexia nervosa
BMI: Body mass index
EAT-26: Eating Attitude Test-26
EDE-Q: Eating Disorders Examination Questionnaire
EDs: Eating disorders
SCOFF: Sick, Control, One Stone, Fat, and Food questionnaire
SCOFF-2.5: Revised version of Sick, Control, One Stone, Fat, and Food questionnaire
SCOFF-O: Original version of Sick, Control, One Stone, Fat, and Food questionnaire
